# Oesophageal reconstruction with a reversed gastric conduit for a complex oesophageal cancer patient: a case report

**DOI:** 10.1186/s12893-022-01630-y

**Published:** 2022-06-11

**Authors:** Yanbo Yang, Lin Ma

**Affiliations:** 1 Department of Thoracic Surgery, West China Hospital, Sichuan University, No. 37, Guoxue Alley, Sichuan 610041 Chengdu, People’s Republic of China; 2 Chest Oncology Institute, West China Hospital, Sichuan University, Chengdu, China; 3 Western China Collaborative Innovation Centre for Early Diagnosis and Multidisciplinary Therapy of Lung Cancer, Sichuan University, Chengdu, China

**Keywords:** Oesophageal reconstruction, Reversed gastric conduit, Oesophageal carcinoma, Pylorus-preserving pancreaticoduodenectomy (PPPD), Gut physiological function

## Abstract

**Background:**

The gastric conduit is the best replacement organ for oesophageal reconstruction, but a reversed gastric conduit (RGC) is rare. Oesophageal reconstruction for oesophageal cancer patients with a previous history of complicated gastrointestinal surgery is rather difficult. Here, we report a case in which oesophageal reconstruction was successfully managed using RGC based solely on the left gastroepiploic artery supply.

**Case presentation:**

A 69-year-old man with oesophageal cancer had a history of endoscopic intestinal polypectomy and pylorus-preserving pancreaticoduodenectomy (PPPD). The right gastroepiploic artery and right gastric artery had been completely severed. The only supply artery that could be used for the gastric conduit was just the left gastroepiploic artery. Because of the complex history of abdominal surgery, we had no choice but to use the RGC to complete the oesophageal reconstruction, in which the gastric conduit was passed reversely through the hiatus to the oesophageal bed and layered end-to-side manual intrathoracic anastomosis with the esophagus. The patient had transient feeding problems with postoperative delayed thoracic stomach emptying but no anastomotic stenosis or thoracic stomach fistula. He was satisfied with his life and had no long-term complications. There was no significant effect on gut physiological function, and RGC could work normally.

**Conclusions:**

Oesophageal reconstruction with RGC is a feasible procedure for complex oesophageal carcinoma that can simplify complicated surgical procedures, has less influence on gut function, is less invasive, and is safe.

## Background

The choice of oesophageal substitute is crucial for oesophageal reconstruction. Oesophageal reconstruction with the stomach after oesophagectomy is the preferred organ and the most commonly accepted standard because of its easy access, elasticity, and comparably ample vascular supply [1, 2]. Although stomach, colon, and jejunum free revascularized grafts are available as potential conduits, a gastric conduit is used [3]. In past studies, a nongastric conduit has been associated with higher morbidity and mortality rates. Moreover, whether the blood supply of the available replacement organ is sufficient is the key to oesophageal reconstruction [4].

In oesophagectomy and reconstruction for treating middle and lower thoracic oesophageal carcinoma, a case of oesophageal cancer with a history of pylorus-preserving pancreaticoduodenectomy (PPPD) and endoscopic intestinal polypectomy placed us in a difficult situation, as follows. The right gastroepiploic artery and right gastric artery had been completely severed in the previous operation. The only supply artery that can be used for the gastric conduit for ER was just the left gastroepiploic artery. The shorter jejunum, intra-abdominal adhesions, and pathological colon forced us to choose to use the RGC to complete the oesophageal reconstruction. Although oesophageal reconstruction with RGC changed the anatomical structure of the gastrointestinal tract and the direction of gastric conduit peristalsis, there was no significant change in gut physiological function.

## Case presentation

**Fig. 1:**
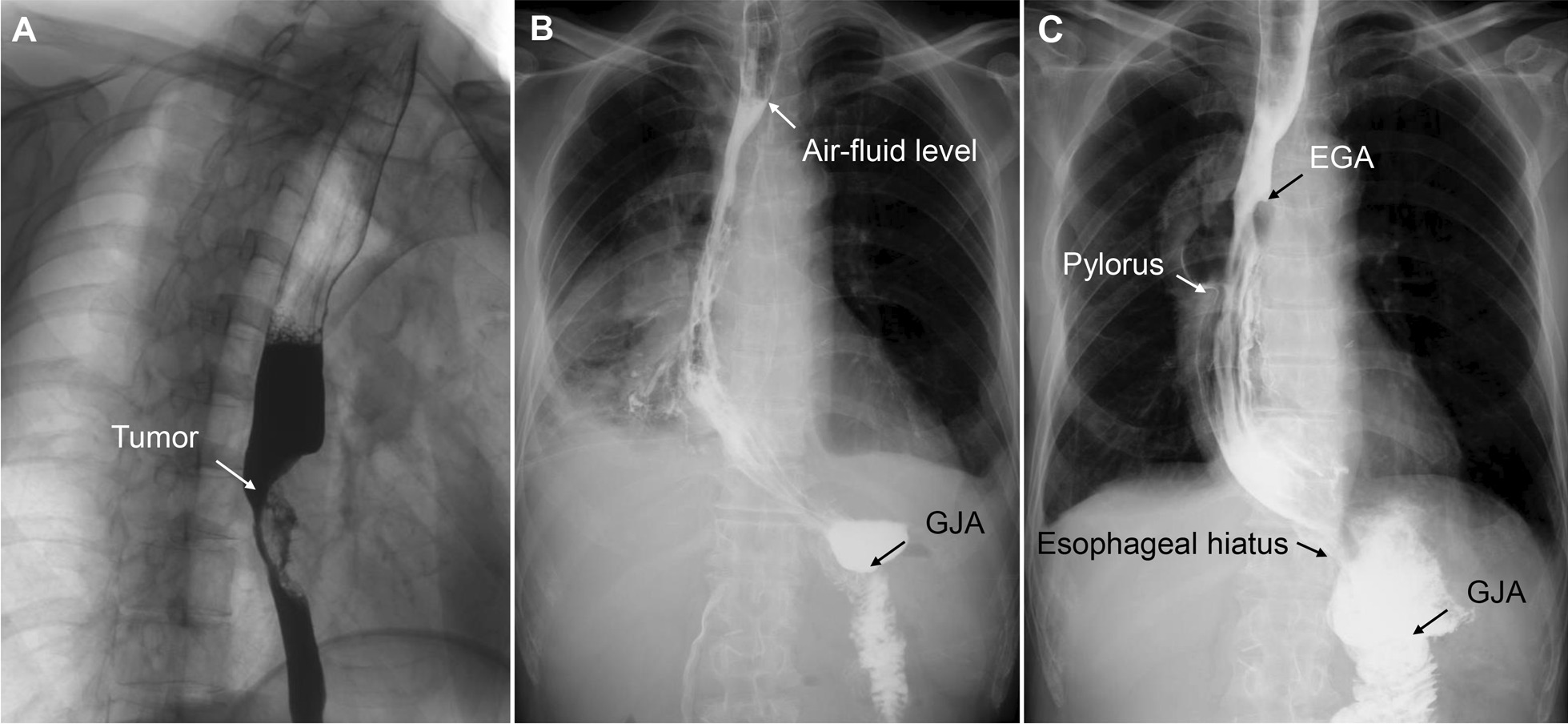
Upper gastrointestinal contrast. **A** Preoperative photograph shows the oesophageal tumour; **B** An air-fluid level above oesophagogastric anastomosis on a postoperative Day 5; **C** Upper gastrointestinal contrast 1 year after surgery. *EGA* oesophagogastric anastomosis, *GJA* gastrojejunal anastomosis

**Fig. 2:**
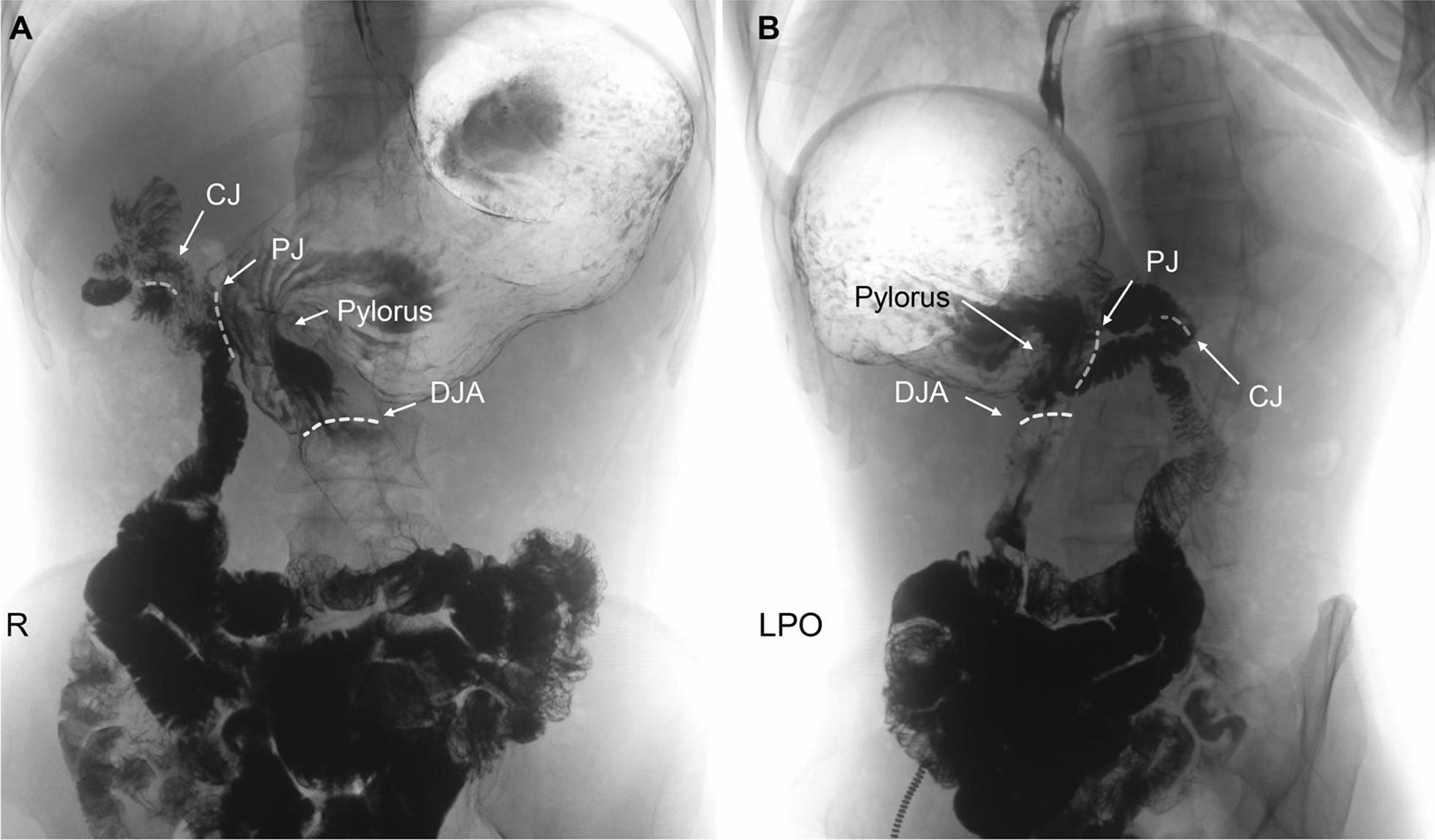
Gastrointestinal contrast for the previous pylorus-preserving pancreaticoduodenectomy. **A** Posterior-anterior; **B** Left posterior oblique. *DJA* duodenal jejunal anastomosis, *CJ* cholangiojejunostomy, *PJ* pancreaticojejunostomy, *R* right, *LPO* left posterior oblique

The patient was a 69-year-old man admitted for nearly 2 months of progressive dysphagia with histories of intestinal polyps and intraductal papillary mucinous neoplasm (IPMN) of the pancreas who underwent endoscopic surgery intestinal polypectomy and laparoscopic PPPD, respectively 9 months ago. Endoscopy showed a tumour in the middle, and lower thoracic oesophagus (29 to 35 cm from the incisors), and a biopsy revealed a squamous cell carcinoma. Further contrast-enhanced computed tomography (CT) and gastrointestinal contrast showed that the tumour was above the inferior pulmonary vein (Fig. 1A), without extraesophageal invasion. Previous gastrointestinal anastomosis of PPPD was illustrated by gastrointestinal contrast (Fig. 2A, B). No evidence of lymphadenopathy or distant metastasis was evaluated by fluorodeoxyglucose-positron emission tomography (FDG-PET). Therefore, the tumor was clinically staged as cT3N0M0 and was considered for primary resection (Ivor-Lewis oesophagectomy).

### Intraoperative exploration and surgical decision

**Fig. 3:**
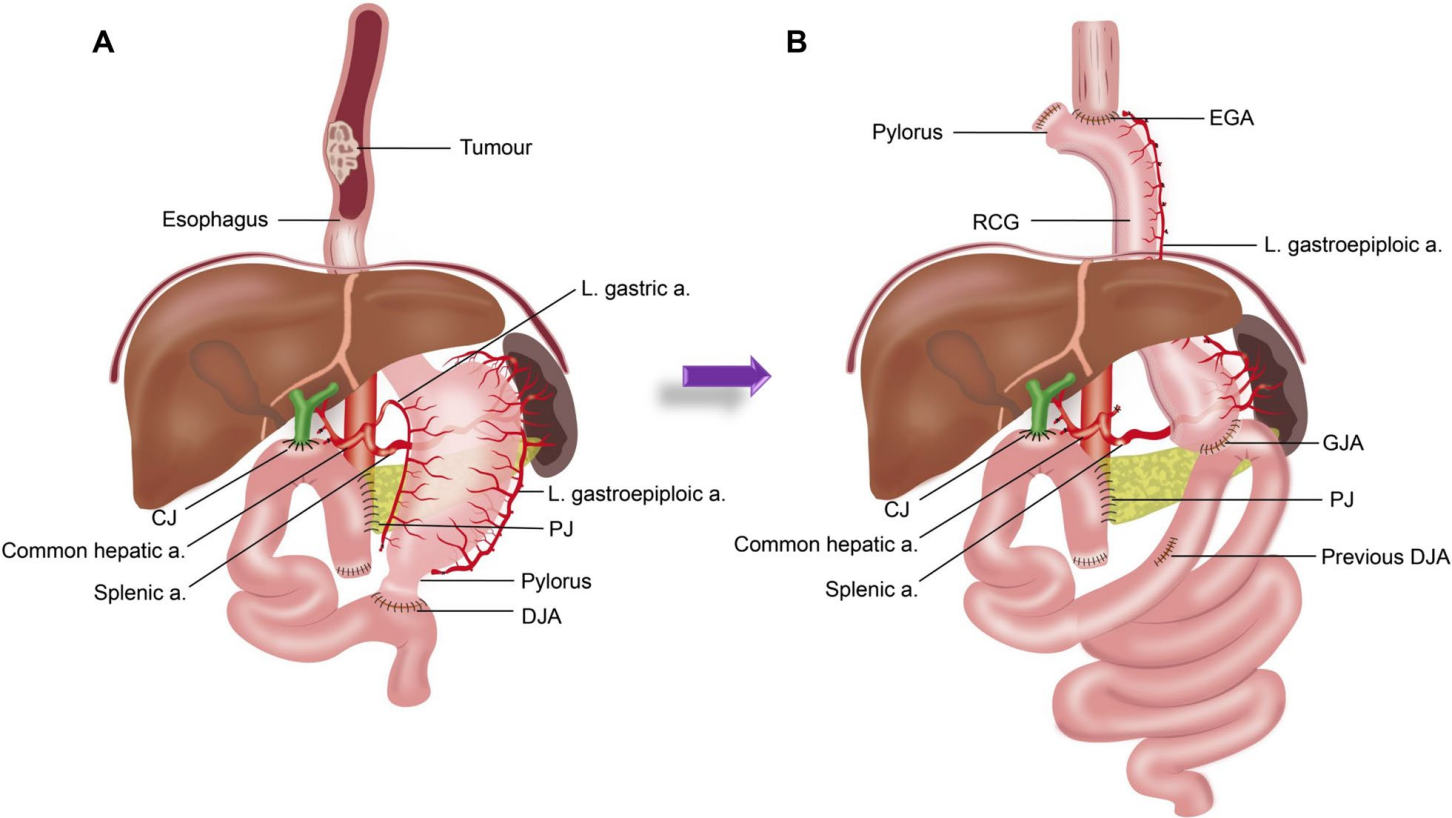
Operative diagrammatic sketch. **A** Diagrammatic sketch of gastrointestinal anastomosis (PPPD) before esophagus reconstruction; **B** Diagrammatic sketch of oesophageal reconstruction with reversed gastric conduit. *RGC* reversed gastric conduit, *EGA* oesophagogastric anastomosis, *GJA* gastrojejunal anastomosis, *DJA* duodenal jejunal anastomosis, *CJ* cholangiojejunostomy, *PJ* pancreaticojejunostomy

Abdominal exploration was performed by median laparotomy, moderate intra-abdominal adhesion around the residual pancreas and near anastomosis, the remaining jejunum and ileum also had adhesions, and the proximal right gastroepiploic artery and right gastric artery were both severed in the previous operation. Fortunately, the vessel arch of the greater curvature was still intact, and the blood supply to the remnant stomach was the left gastroepiploic artery, left gastric artery, posterior gastric artery and short gastric vessels (as shown in Fig. 3A). Because of colonic polyps, short jejunum (due to previous operation and digestive tract reconstruction) and intestinal adhesion, we realised that they could not be used as an oesophageal substitute except for remnant stomach.

### Surgical procedure

The remnant stomach was then mobilised by excising the cardia and lesser curvature stomach, including the left gastric artery, removing the duodenal jejunal anastomosis and dividing the gastrohepatic omentum. After complete mobilisation of the stomach, a 3-cm-wide gastric conduit was created using closer linear cutting (Ethicon ECHELON + Stapler PSEE60A). Next, a new side-to-side anastomosis was reconstructed between the gastric fundus and jejunum, and no feeding jejunostomy was performed. The left gastroepiploic artery, posterior gastric artery and short gastric vessels were reserved in the original location to ensure the blood supply of the thoracic stomach. Hence, the left gastroepiploic artery became the only source of blood supply to the gastric conduit (Fig. 3B).

Thoracotomy was performed via a posterolateral incision at the right fifth intercostal. The oesophagus was dissected from the oesophagogastric junction to the level of the azygos vein arch. Complete resection of the oesophageal tumour was achieved. Lymph node dissection was routinely performed at the time of resection, which include Stations 2R, 4R, 4 L, 7, 8U, 8 M, 8Lo, 9R, 10R, 15, 16 and 17. The gastric conduit was passed reversely through the hiatus to the oesophageal bed and layered end-to-side manual intrathoracic anastomosis with the oesophagus (Fig. 3B). Microscopic examination revealed a moderately differentiated squamous cell carcinoma without evidence of lymph node metastasis at any station (pT3N0M0 G2, stage IIB) and a negative surgical margin (R0).

### Postoperative management and follow-up

After surgery, the patient received gastrointestinal decompression and 5 days total parenteral nutrition support. He was started on a liquid diet on postoperative Day 6 and a soft blended diet on Day 10. Thoracic stomach emptying was delayed, but no anastomotic stenosis or thoracic stomach fistula was documented by upper gastrointestinal contrast on a postoperative Day 5 (Fig. 1B). After liquid diet intake for 3 days, symptoms from delayed gastric emptying largely disappeared, and the patient was discharged from the hospital on a postoperative Day 12 with no complications. Follow-up at the third month after the operation showed that the patient was satisfied with his life and had no complications. No thoracic stomach emptying was delayed, anastomotic stenosis, thoracic stomach dilatation or conduit redundancy was observed at the one-year-follow-up (Fig. 1C), and no gastrointestinal dysfunction or anemia was observed.

## Discussion and conclusions

There has been an increase in the number of patients with double cancers co-occurring or metachronously in recent years. Most of them have found periampullary cancers, such as pancreatic head cancer, bile duct cancer, and cancer of the papilla Vater, after oesophagectomy for oesophageal carcinoma [5]. However, cases of oesophagectomy and reconstruction for oesophageal carcinoma after pancreatoduodenectomy or PPPD for pancreatic head cancer are rare. Pancreatoduodenectomy or PPPD is the standard surgery for resectable periampullary cancer. It involves resectioning the gastroduodenal artery and its branches, such as the right gastroepiploic artery to allow for complete dissection of lymph vessels and nodes [5]. When oesophageal cancer occurs in patients who need to reconstruct the oesophagus only by the gastric conduit because other organs are not available, the resected gastroduodenal artery or gastroepiploic artery, which is the major vessel in the greater curvature of the stomach, could lead to serious complications (such as anastomotic leakage and gastric conduit necrosis) due to insufficient blood supply to the gastric conduit [4]. Both the colon and supercharged pedicled jejunum are acceptable options for ER when the stomach is unavailable [6]. Unfortunately, a preoperative evaluation of the colon and jejunum were not available for the present case due to previous operations for colonic polyps, shortened jejunum, and intestinal adhesions. Moreover, the longer operative time required for colonic or jejunal mobilization and the additional anastomosis increase surgical stress and postoperative complications [7].

Conventional reversed gastric tube oesophagoplasty is mainly required to treat oesophageal atresia and caustic oesophageal strictures in infants and children [8]. However, a small amount was applied to failure cases in the treatment of colon, jejunum and prosthetic interpositions [9]. These gastric tubes were made of a part of the greater curvature gastric with gastroepiploic vessels and the lesser curvature gastric. Most of the gastric body still retained the original position of the abdominal cavity, and was not resected. Reversed gastric tubes are rare for oesophagogastrectomy and primary reconstruction in the treatment of oesophageal cancer. By studying the gastrointestinal contrast and reviewing the history of previous abdomen surgery, we recognised that we had no choice but just the gastric conduit, which had been pulled up reversely through the oesophageal hiatus to the oesophageal bed to complete oesophageal reconstruction. This method could also simplify surgical procedures and shorten the operative time. The patient had transient postoperative delayed thoracic gastric emptying without reflux, probably because the thoracic stomach still has weak receptive relaxation function despite the cardia, lesser curvature gastric body and vagus nerve being excised. However, no prokinetic agents were applied, and the patient recovered within two weeks. The peristaltic function of RGC was weakened after the operation; they were mainly used as conduits for transporting chyme, and other functions, such as secretion, were weakened or even disappeared. RGC could generally work without any long-term complications.

Usually, the gastric conduit is supplied by two main arteries, the right gastroepiploic artery and the right gastric artery. Cancerous, atherosclerotic or surgical involvement of the right gastroepiploic artery is a contraindication for transthoracic and transhiatal oesophagectomies [10]. However, the gastric conduit we presented was supplied mainly by the left gastroepiploic artery, which has been shown to have sufficient blood supply for the gastric conduit after ligation of the other gastric arteries intraoperatively. Procedures involving a gastric conduit or the whole stomach have become widely accepted as standard replacement organs for ER for a long time, however, the most feared complications are anastomotic leakage and gastric conduit necrosis. We successfully reconstructed the oesophagus using RGC, a nontraditional reversed gastric conduit. The only significant source of blood supply to the thoracic stomach was the left gastroepiploic artery, which was reasonably competent to supply the RGC. No long-term postoperative complications occurred, and patient was satisfied with his life after the operation. This is the first reported case of successful oesophageal reconstruction with RGC based solely on the left gastroepiploic artery to treat an oesophageal carcinoma patient.

In conclusion, oesophageal reconstruction with RGC is a feasible procedure for complex oesophageal carcinoma that can simplify complicated surgical procedures, has less influence on gut function, is less invasive, is relatively safe and is less time-consuming since no further reconstruction of the alimentary tract or the vascular system is applied.

## Data Availability

The datasets used and/or analysed during the current study are available from the corresponding author on reasonable request.

## Abbreviations

RGC: Reversed gastric conduit
PPPD: Pylorus-preserving pancreaticoduodenectomy
IPMN: Intraductal papillary mucinous neoplasm
